# Correction to “A p21‐GFP zebrafish model of senescence for rapid testing of senolytics in vivo”

**DOI:** 10.1111/acel.14088

**Published:** 2024-01-29

Samir Morsli, Catarina M. Henriques, Pamela S. Ellis, Heather Mortiboys, Sarah Baxendale, Catherine A. Loynes, Stephen A. Renshaw, Ilaria Bellantuono. 2023;22:6:e13835.

In Figure 4g, the bottom left hand graph is a duplication of the graph on the bottom right. Below is the version of this figure with the correct graphs.

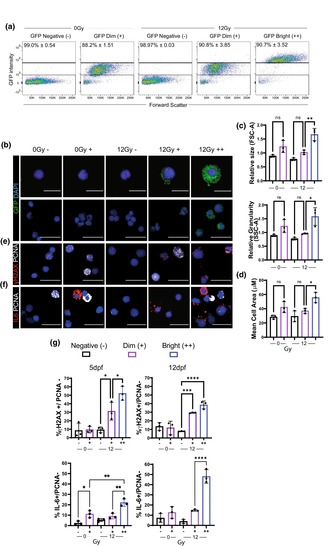

We apologize for this error.

