# Supplementary figures and images for: A p21‐GFP zebrafish model of senescence for rapid testing of senolytics in vivo
Source: Aging Cell. 2023 Apr 11;22(6):e13835. doi: 10.1111/acel.13835 (PMC10265157; doi:10.1111/acel.13835)

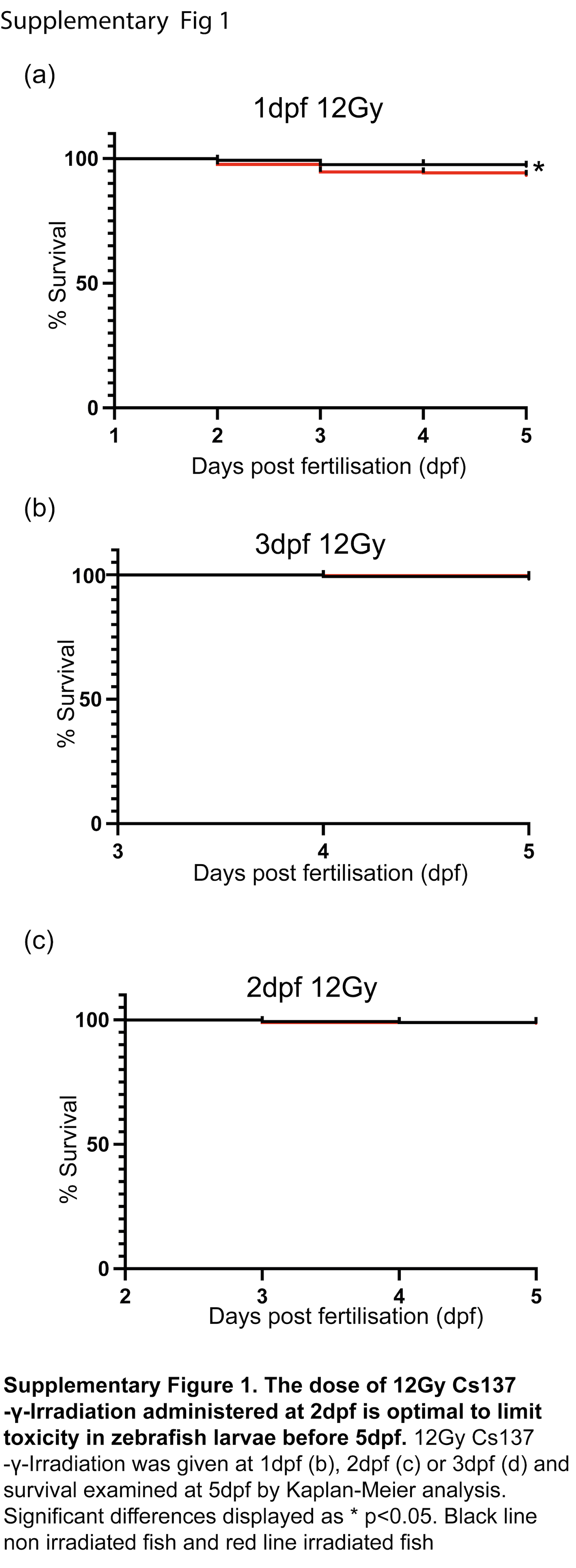

Supplement: Supplementary file 1 — Figure S1 [file ACEL-22-e13835-s003.png]

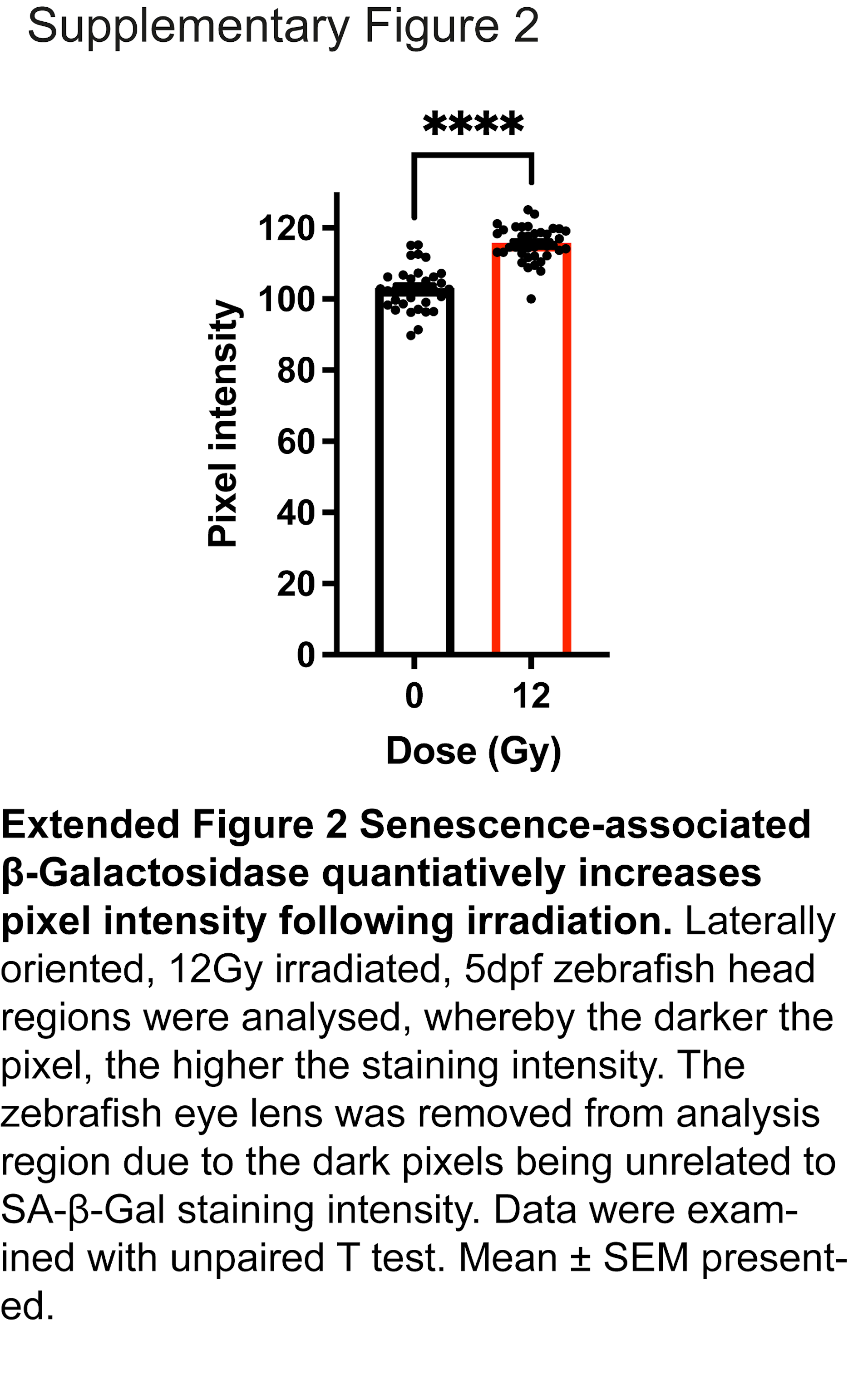

Supplement: Supplementary file 2 — Figure S2 [file ACEL-22-e13835-s001.png]

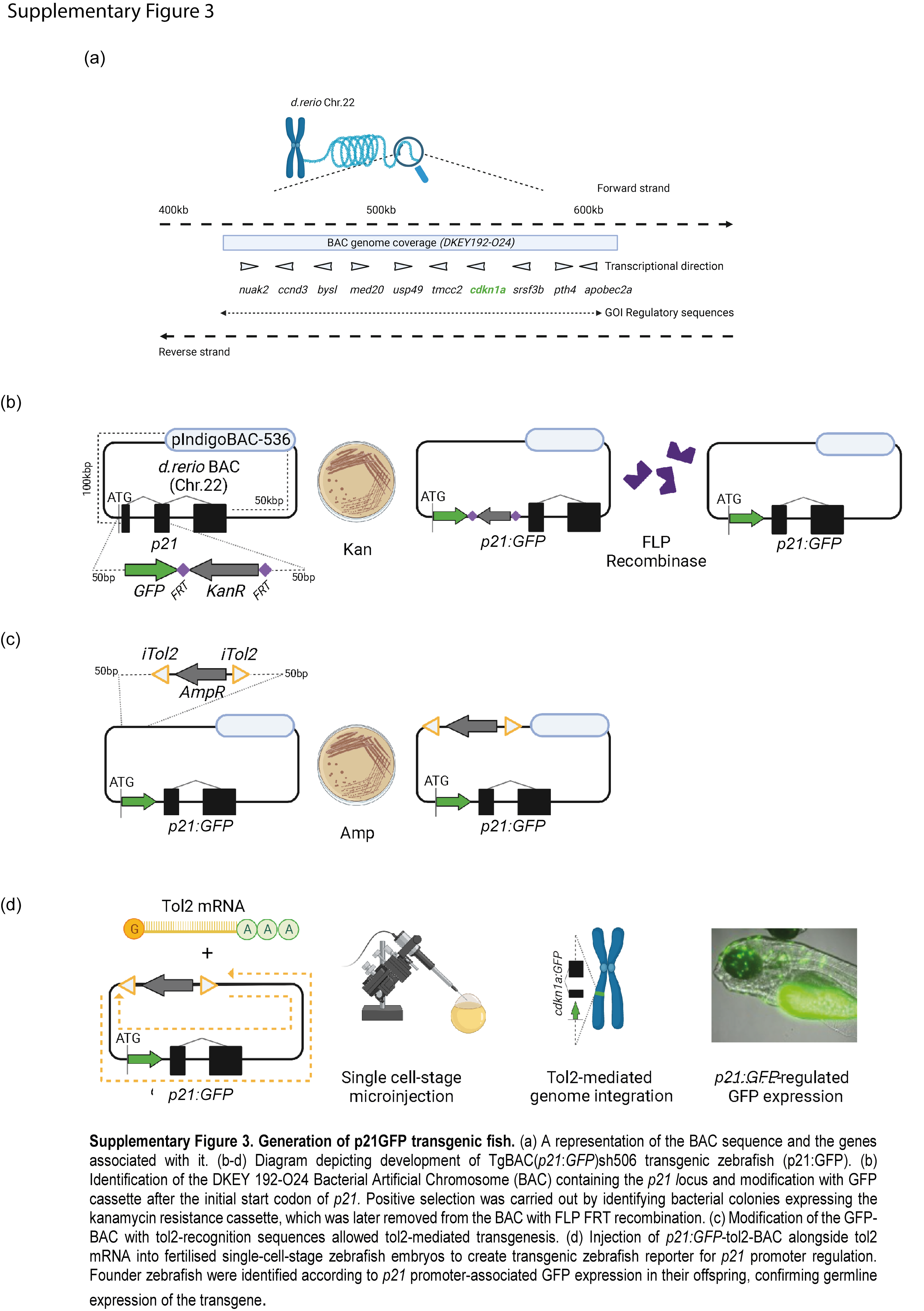

Supplement: Supplementary file 3 — Figure S3 [file ACEL-22-e13835-s002.png]

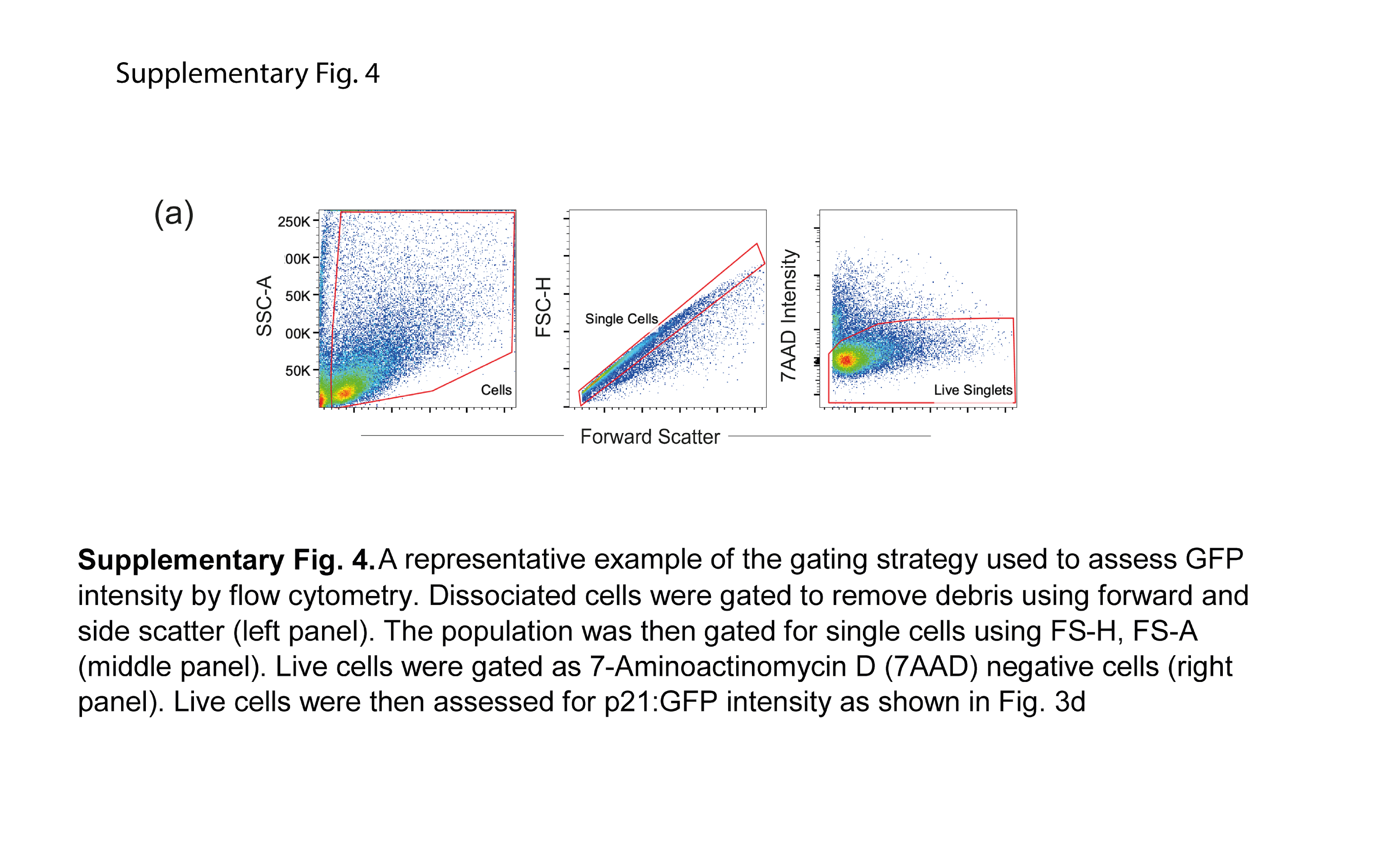

Supplement: Supplementary file 4 — Figure S4 [file ACEL-22-e13835-s004.png]

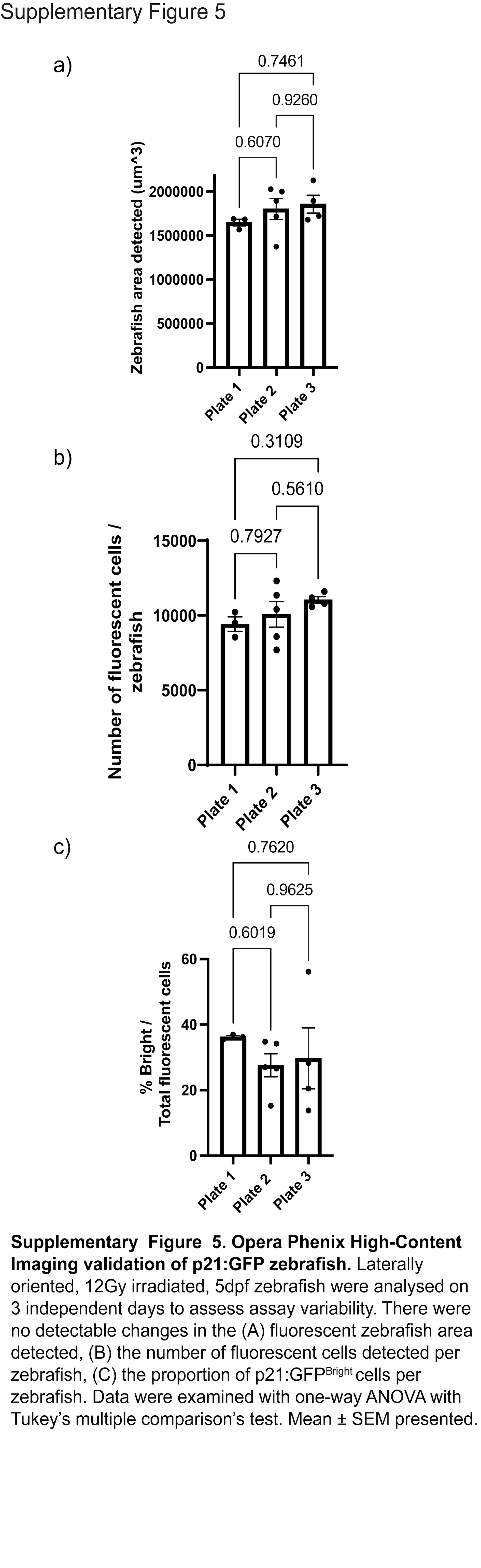

Supplement: Supplementary file 5 — Figure S5 [file ACEL-22-e13835-s005.png]
